# Supplementary material for: Genomics-Based Identification of Microorganisms in Human Ocular Body Fluid
Source: Sci Rep. 2018 Mar 7;8:4126. doi: 10.1038/s41598-018-22416-4 (PMC5841358; doi:10.1038/s41598-018-22416-4)
Supplement: Supplementary file 1 — Supplementary Figures & Methods [file 41598_2018_22416_MOESM1_ESM.pdf]

Supplementary Figures & Methods

Genomics-Based Identification of Microorganisms in Human Ocular Body Fluid

[1]Philipp Kirstahler, [2]Søren Solborg Bjerrum, [3]Alice Friis-Møller, [2]Morten la Cour, [1]Frank M. Aarestrup, [3,4]Henrik Westh, and [1]Sünje Johanna Pamp[1*]

[1]Research Group for Genomic Epidemiology, Technical University of Denmark, Kgs. Lyngby, Denmark; [2]Department of Ophthalmology, Rigshospitalet, Copenhagen, Denmark; [3]Department of Clinical Microbiology, Hvidovre Hospital, Copenhagen, Denmark; [4]Department of Clinical Medicine, Faculty of Health and Medical Sciences, University of Copenhagen, Copenhagen, Denmark.

*corresponding author sjpa@food.dtu.dk.

Supplementary Figures

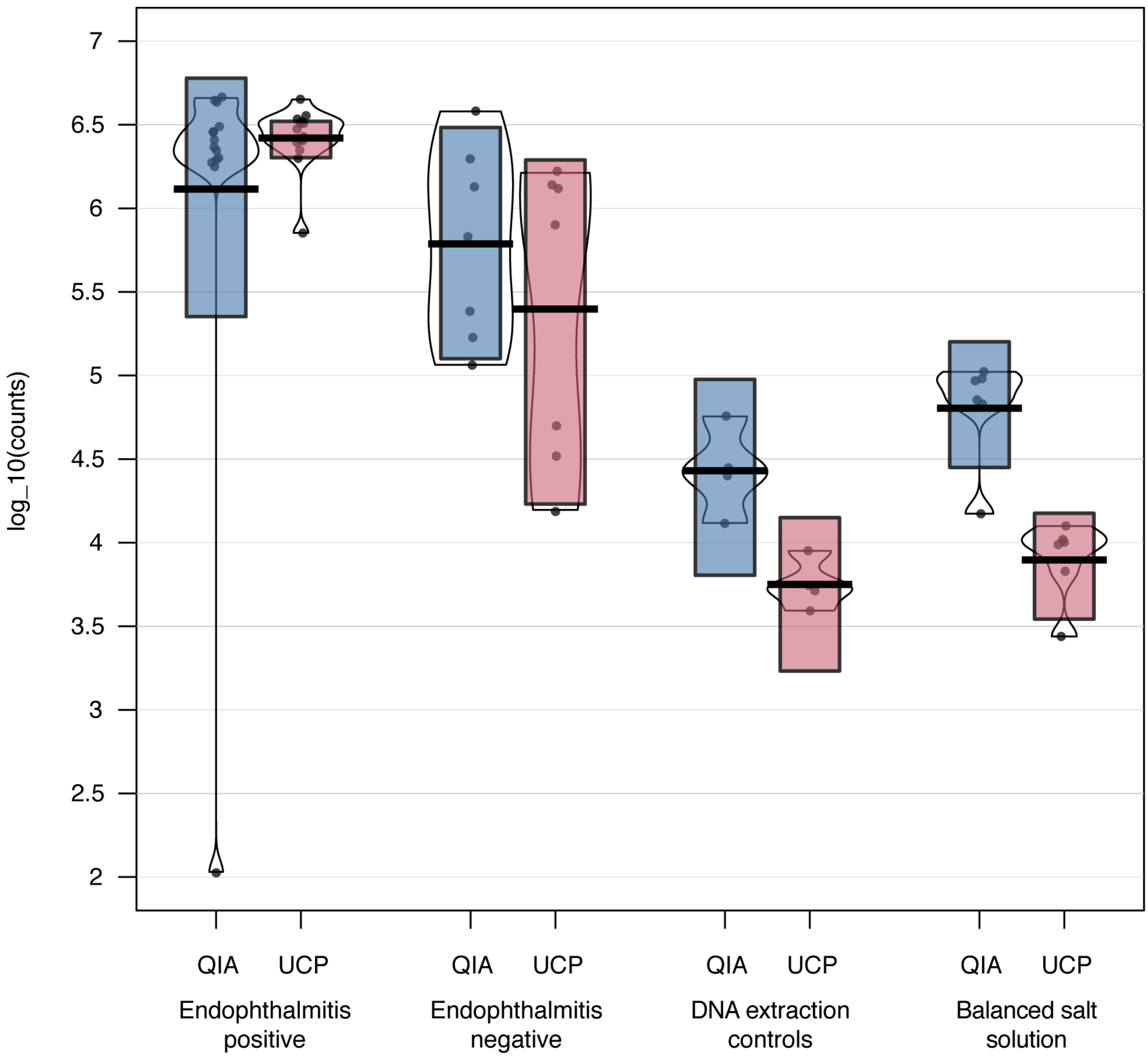

Figure S1: Read counts per sample group and DNA isolation method obtained using shotgun metagenomic sequencing. Number of reads obtained using metagenomic sequencing (MiSeq) that passed the quality trimming and filtering. The samples are listed according to sample group (endophthalmitis negative, endophthalmitis positive, DNA extraction controls, balanced salt solution) and DNA extraction method (QIAamp DNA Mini Kit [QIA], QIAamp UCP Pathogen Mini kit [UCP]). The points in the plot represent the individual samples, the bean represents the smoothed reads count density based on the samples, the colored rectangle indicates the 95% highest density interval, and the horizontal bar indicates the central tendency. For results from a statistical analysis see https://figshare.com/s/fb84c864b9b49205db3f.

A)

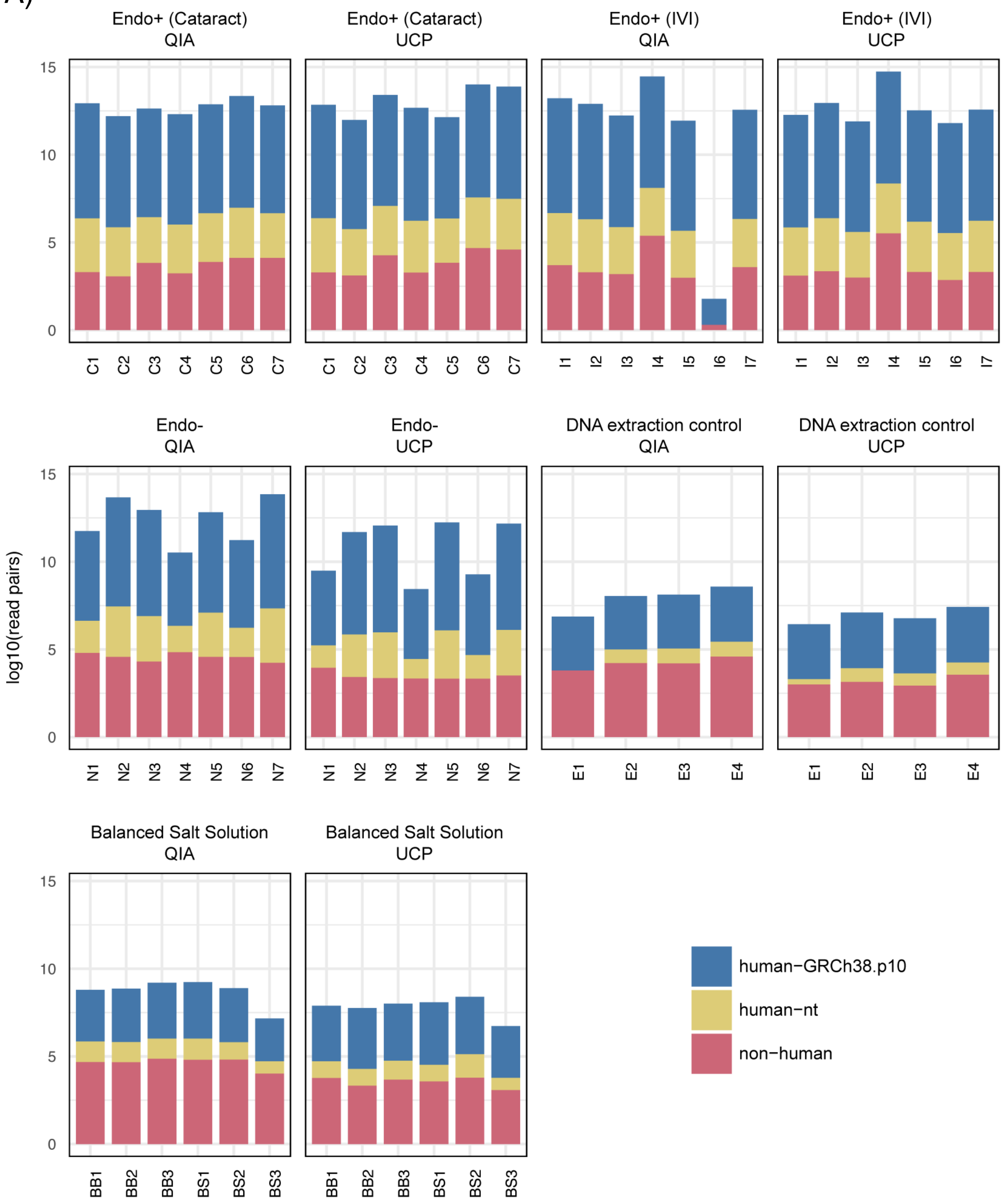
Endo+ (Cataract) QIA
Endo+ (Cataract) UCP
Endo+ (IVI) QIA
Endo+ (IVI) UCP
Endo- QIA
Endo- UCP
DNA extraction control QIA
DNA extraction control UCP
Balanced Salt Solution QIA
Balanced Salt Solution UCP
log10(read pairs)
0
5
10
15
C1 C2 C3 C4 C5 C6 C7
I1 I2 I3 I4 I5 I6 I7
N1 N2 N3 N4 N5 N6 N7
E1 E2 E3 E4
BB1 BB2 BB3 BS1 BS2 BS3
human−GRCh38.p10
human−nt
non−human

B)

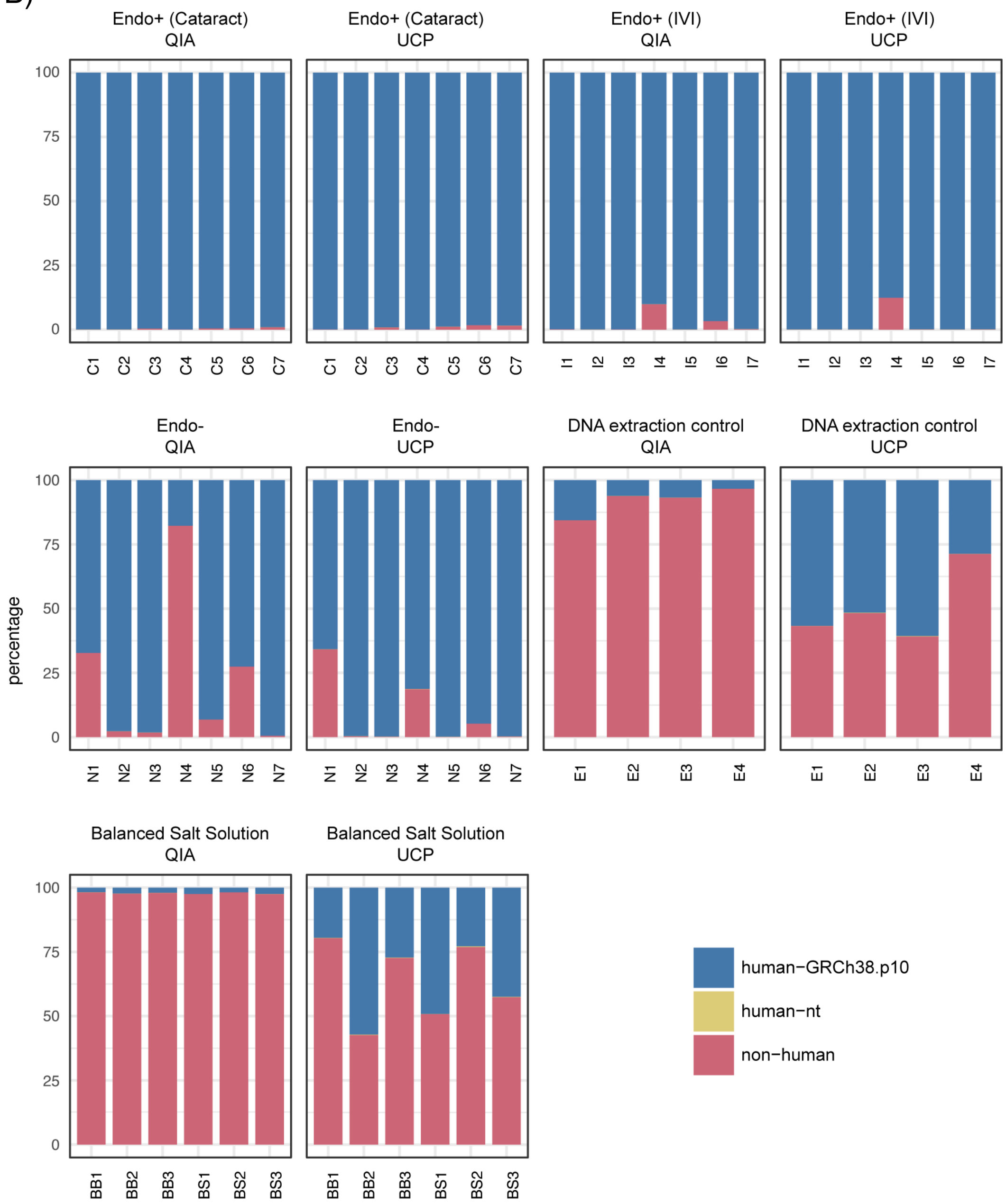

Figure S2: Human-affiliated reads in the metagenomic sequencing data. The reads from endophthalmitis-negative patients, endophthalmitis-positive patients (post cataract, and post intravitreal injection (IVI)), as well as the DNA extraction controls, and balanced salt solution samples were mapped against the human genome assembly GRCh38.p10. The non-mapped reads were aligned using BLASTn to the non-redundant nucleotide collection nt database from NCBI. Reads mapped to GRCh38.p10 (blue), reads aligned to human genome sequences in NCBI nt database (yellow), and reads that were not affiliated with any human genome sequences in the two databases (red). Figure A) displays the log^{10} counts of read pairs and Figure B) displays the relative abundance of human and non-human affiliated reads.

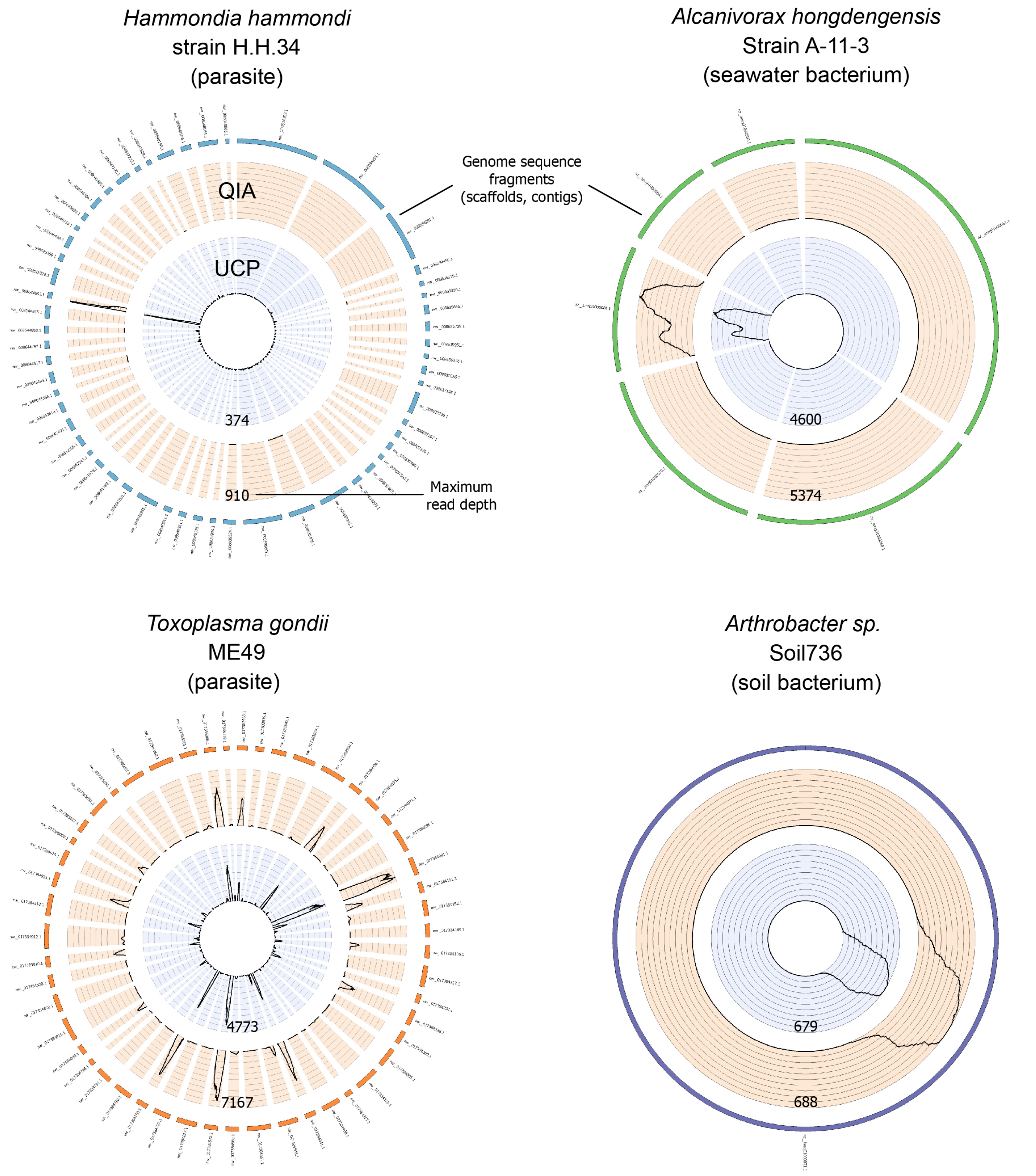

Figure S3: Selected genomes from public databases containing ambiguous (putative contaminant) DNA sequences. Reads of this study mapped to specific genome sequence fragments (scaffolds, contigs) of the genomes of *Hammondia hammondi* strain H.H.34 (GCF_000258005.1), *Alcanivorax hongdengensis* Strain A-11-3 (AMRJ00000000), *Toxoplasma gondii* ME49 (GCF_000006565.2), and *Arthrobacter* sp. Soil736 (GCF_001428005.1). The most outer circle displays genome sequence fragments (scaffolds, contigs), and the Top50 contigs with at least a minimum coverage of 5 per nucleotide position are shown. When these contigs were aligned to the nucleotide collection nt (NCBI), their Top10 hits included in most cases human genomic sequences and in other

cases bacterial sequences (see Supplementary Table S3). The orange and blue inner circles display the depth of mapped reads originating from the samples that were extracted with the QIA and UCP DNA extraction methods, respectively. The corresponding maximum read depth is indicated at the bottom of the ring. For a detailed list of scaffolds and contigs predicted to be ambiguous in the 5750 public microbial genomes examined in this study, see https://figshare.com/s/c42158cdee23f25489cd.

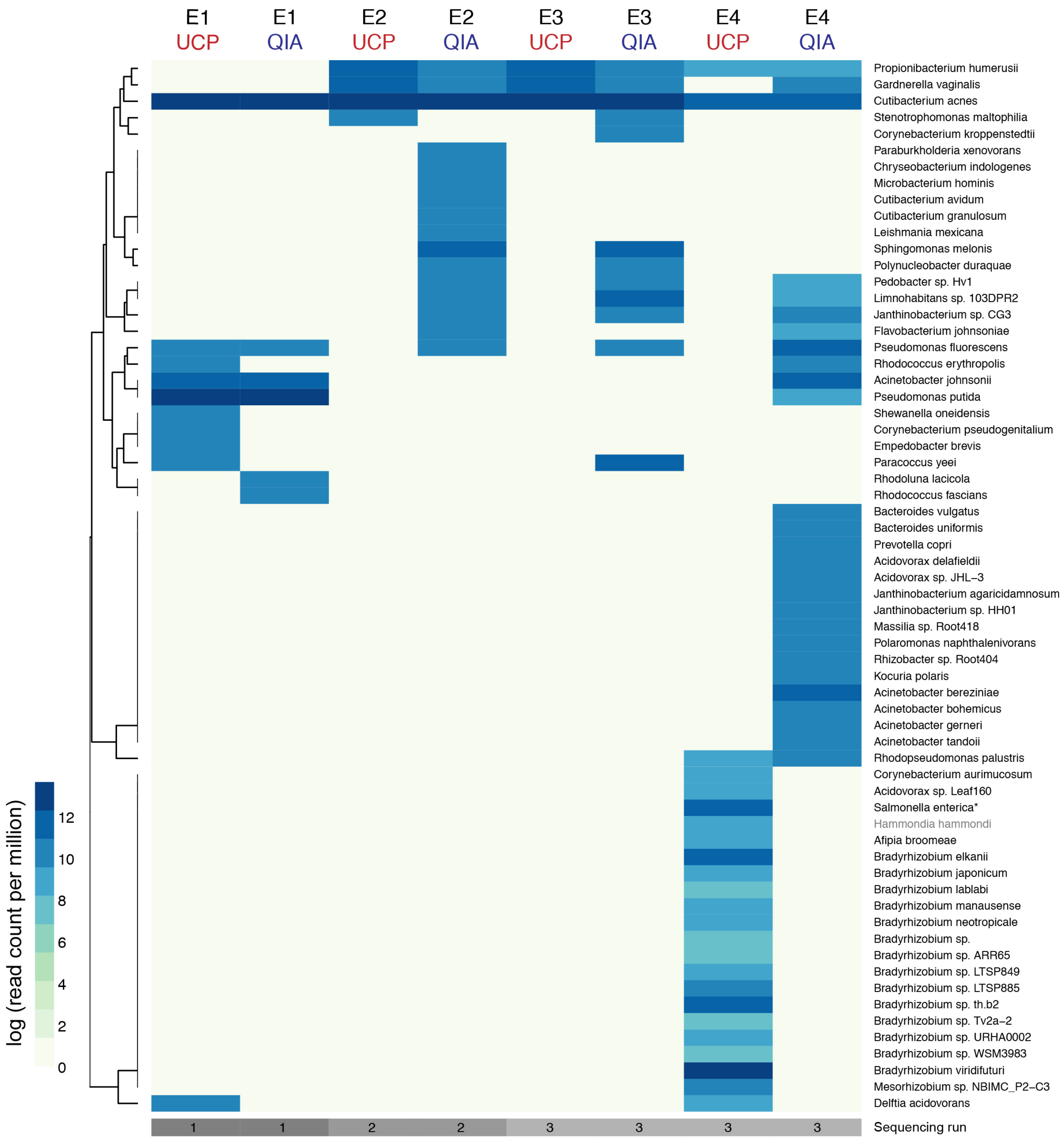

Figure S4: Abundance of different organisms in DNA extraction (blank) control samples. The Top20 most abundant organisms per sample for DNA extraction control E1 to E4, extracted at four different time points with the QIAamp DNA Mini Kit (QIA) and QIAamp UCP Pathogen Mini kit (UCP) respectively, are displayed. In some samples fewer than 20 different microorganisms were detected. Read counts are presented as counts per million. See Supplementary Table S5 for a complete list of contaminant taxa detected in the individual samples. *Salmonella enterica* (labeled with an asterisk) may originate from carry-

over contamination during sequencing as it was sequenced on the same Illumina MiSeq machine in a prior sequencing run. *Hammondia hammondi* (grey) may be a false positive, as its public reference genome sequence contained ambiguous (potential contaminant) contigs or scaffolds (see Supplementary Table S3).

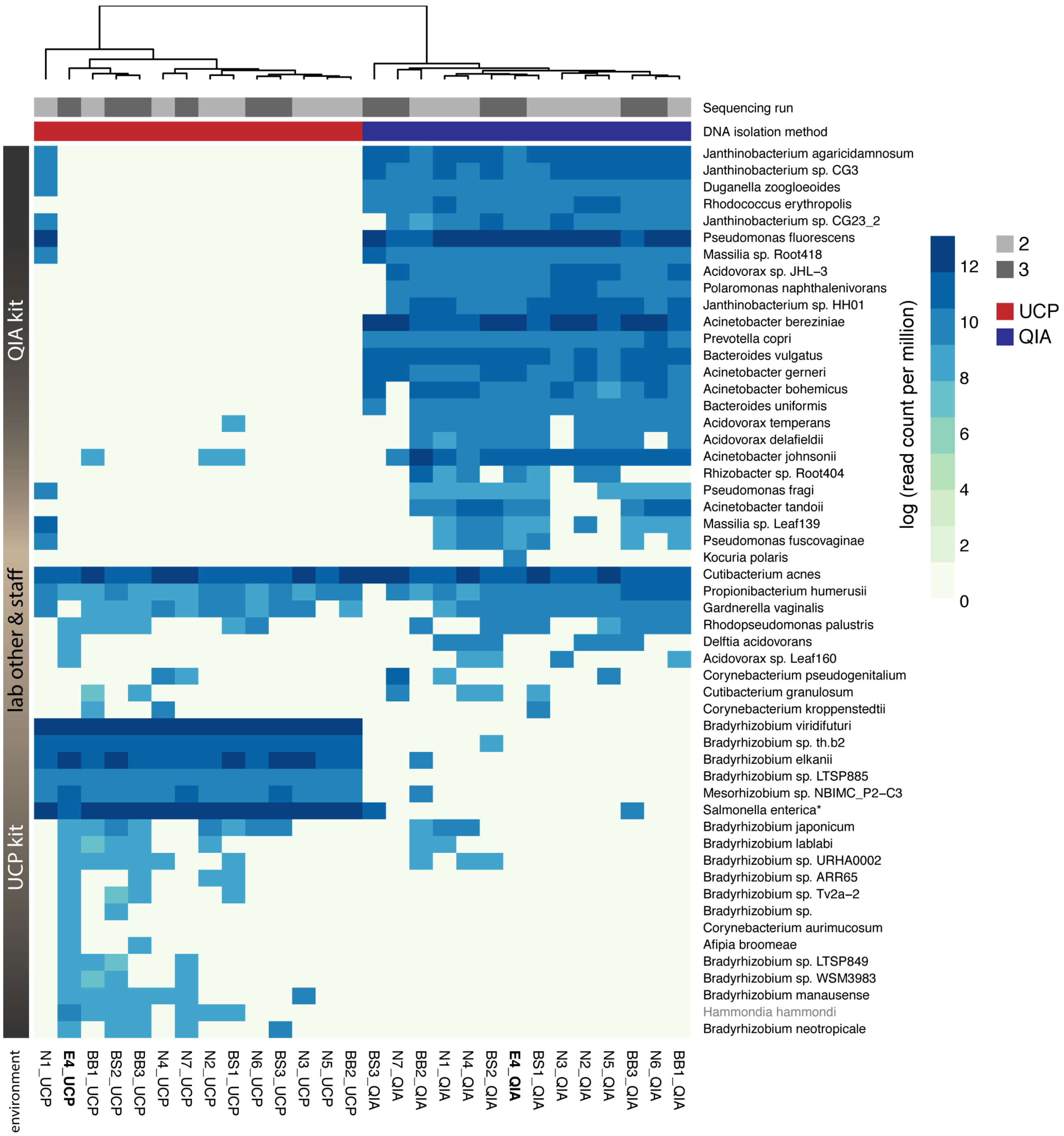

Figure S5: Abundance of taxa in endophthalmitis-negative, balanced salt solution, and associated DNA extraction (blank) control samples. The 20 most abundant taxa per sample group are displayed, i.e. the Top20 organisms for E1_QIA, E1_UCP, endophthalmitis-negative QIA (N1-7_QIA), endophthalmitis-negative UCP (N1-7_UCP), balanced salt solution QIA (BB1-3_QIA, BS1-3_QIA), and balanced salt solution UCP (BB1-3_UCP, BS1-3_UCP), respectively. The abundance of taxa is displayed as counts per million. Three main groups of organisms emerge: organisms that are predominantly found in QIA-related samples, UCP-related samples, or both sample groups. The latter group may be environmental organisms originating from the laboratory equipment and supplies, and/or laboratory staff. Some taxa may also originate from carry-over contamination during sequencing as the following species were sequenced on the same Illumina MiSeq machine

in respective prior sequencing runs: *Salmonella enterica* (labeled with an asterisk), and *Plasmodium falciparum* and *Escherichia coli* (not displayed, as not among the Top20 organisms). *Hammondia hammondi* (grey) may be a false positive, as its public genome sequence contained ambiguous (potential contaminant) contigs or scaffolds (see Supplementary Table S3). It should be noted that fewer microorganisms were detected in the DNA extraction controls E1 – E3 for both kits (not displayed).

Extended supplemental material

Further material, in addition to the Electronic supplemental material available from the journal website, is available through the following repositories:

I) *Figures and Tables*

https://figshare.com/projects/Genomics-Based_Identification_of_Microorganisms_in_Human_Ocular_Body_Fluid/21038
(umbrella project)

Classification of microbial genomes against human reference genome - (Item A)
https://figshare.com/s/5cc8f045347a93743739

The effect of contaminated reference genomes in clinical metagenomics (Item B)
https://figshare.com/s/a282670f1405eae232df

Ambiguous sequences in public microbial genomes - Overview - (Item C)
https://figshare.com/s/045b1252bd7555b50ef0

Ambiguous sequences in public microbial genomes - Sequence List - (Item D)
https://figshare.com/s/c42158cdee23f25489cd

Ambiguous sequences at different cut off levels - (Item E)
https://figshare.com/s/b2db263f05db3b571aed

Selected contaminant and non-contaminant organisms - (Item F)
https://figshare.com/s/a4fd9d84260e8456ab72

Read counts for most abundant microbial agent in patient samples - (Item G)
https://figshare.com/s/5feabfad1d8c495bf7a3

Targeted PCR on vitreous DNA - (Item H)
https://figshare.com/s/0e8a98f436f07efc4dd5

Mapping of reads to genomes - (Item I)
https://figshare.com/s/c2ce2d32daf25db54904

Phenotypic antibiotic susceptibility - (Item J)
https://figshare.com/s/e579abea97dfc8c77a6a

Virus genomes in extended database - (Item K)
https://figshare.com/s/b040289827b79d3a60df

Bacteriophages/viruses in endophthalmitis and control samples - (Item L)
https://figshare.com/s/ff0527509828d1529ad9

Video Summary of Kirstahler et al. - (Item M)

https://figshare.com/s/38fe043f6a8ef1710444

Guidelines for Metagenomic (Microbiome) Sequencing Projects - (Item N)
https://figshare.com/s/2a0709b1f0c5e18754df

Comparison between QIAamp DNA Mini Kit [QIA], QIAamp UCP Pathogen Mini kit [UCP] – (Item O)
https://figshare.com/s/fb84c864b9b49205db3f

Comparison of bioinformatics workflows – (Item P)
https://figshare.com/s/07dfa4f99e663fc79164

II) *Code for the creation of the curated microbial reference genome database*
https://github.com/philDTU/endoPublication

III) *Curated microbial reference genome sequences*
ftp://ftp.cbs.dtu.dk/public//CGE/databases/CuratedGenomes

Supplementary Methods

Isolation of DNA from complex samples

As there was no standardized protocol for the extraction of DNA from vitreous prior to this study, we analysed each sample using two different DNA isolation procedures. To avoid freeze-thaw cycles, DNA was extracted from frozen vitreous that was thawed on ice, on the same day as the 2nd cultivation was performed. We isolated DNA from the vitreous fluid as well as balanced salt solution samples using two procedures, i) the QIAamp DNA Mini Kit (51304, Qiagen), and ii) the QIAamp UCP Pathogen Mini Kit (50214, Qiagen). In a pilot experiment, we extracted DNA from vitreous fluid using three procedures, i) the QIAamp DNA Mini Kit, ii) QIAamp UCP Pathogen Mini Kit, and iii) QIAamp UCP Pathogen Mini Kit + bead beating step. The microbiome profile resulting from procedure i) and iii) was most similar to each other, and hence we conducted the present study with the two complementing DNA isolation procedures i) and ii). We have previously attempted to remove human DNA from human body fluid samples using a kit from Molzym, with no success, and hence we did not attempt to remove human DNA using this procedure on the limited sample volume we had available for this study.
For each round of DNA isolation, one extraction control (blank) was included that did not include any sample input, and was processed in the same way as the complex sample. One extraction control (blank) was included for 12-14 study samples (more samples were extracted than sequenced in the current study). i) DNA was isolated from 200 µl vitreous body using the QIAamp DNA Mini Kit according to the manufacturer's protocol "DNA Purification from Blood or Body Fluids" with minor modifications. The DNA was eluted in 100 µl AE buffer and stored at -20°C until further use. ii) DNA was isolated from 200 µl vitreous body using the QIAamp UCP Pathogen Mini Kit according to the manufacturer's protocol "Pretreatment of Microbial DNA from Biological Fluids or Cultures" and "Sample Prep Spin Protocol" with minor modifications. The sample pellet was resuspended in 400 µl ATL buffer, and the DNA was eluted in 50 µl AE buffer and stored at -20°C until further use. The DNA concentration was determined using Qubit® dsDNA High Sensitivity Assay Kit on a Qubit® 2.0 Fluorometer (Invitrogen, Carlsbad, CA).

Metagenomic sequencing data analysis

1. *Quality trimming and filtering*
Adapter sequences were filtered out and poor quality bases were removed from the end of the read using BBDuk of BBMap version 35.82 (http://jgi.doe.gov/data-and-tools/bbtools/). The minimal read length was set to 75 bases and the phread score to 20. In addition, reads with low complexity were removed using an entropy value of 0.7. Only intact pairs where kept for further analysis; i.e. both reads of a pair were removed also if one did not fulfil the quality criteria.

2. *Removal of human reads*
We employed a 2-step filter approach for a thorough removal of human reads. In a first step, we mapped all high-quality reads against the reference genome GRCh38.p10 (GCF_000001405.36) using BBDuk with minimal identity set to 0.65, and through which the majority of human-affiliated reads was removed (Supplementary Figure S2). However, because of human genetic individuality not all human DNA sequences are represented in

the single human reference genome sequence. In a second step, we therefore aligned all unmapped reads to a precompiled non-redundant nucleotide collection (nt) database from NCBI (downloaded 27.01.2017) using BLASTn of BLAST version 2.6.0 [1]. We removed all reads that had aligned to human DNA sequences with a minimal e-value of 1e-6.

3. Detection of ambiguous sequences & *creation of curated microbial genome databases*
Detection of ambiguous sequences: We discovered in our analysis of patient samples that certain microbial reference genomes accumulated reads (e.g. *Alcanivorax hongdengensis*, *Hammondia hammondi*). We did not expect these particular organisms to be present in the eye in high numbers, and we could not explain this phenomenon with human sequence contamination only. We noticed that the reads were mainly recruited to specific contigs, scaffolds, or particular genomic regions in the particular organisms, and that mostly small sequence fragments (e.g. small contigs) were affected, or short regions within larger genomic fragments that were in close proximity to stretches of ambiguous base calls (N's). To get a better understanding, we split the microbial reference sequences (archaea, bacteria, fungi, protozoa from NCBI RefSeq database (20.12.2016)) at stretches of 10 Ns or more into separated contigs. After splitting, contigs with less than 100 bases where removed. A total of 761,870 sequences below 10 kb (these were original contigs and scaffolds, and contigs originating from the split at N's) where then aligned against the non-redundant nucleotide collection (nt) database from NCBI using BLASTn with a minimal e-value of 1e-6. [The 10 kb as threshold for short sequences was selected for computational reasons and because most sequences with a high kraken label score, when classifying the microbial genomes of the database against the human reference, were below 10 kb (https://figshare.com/s/5cc8f045347a93743739)]. Only sequences with a best hit outside of their own genus and that covered the query sequence to at least 70% where considered ambiguous. The best hit for a contig was determined based on the bitscore of the alignment. An overview and sequence list of the 70,478 ambiguous contigs is available from figshare at https://figshare.com/s/045b1252bd7555b50ef0 and https://figshare.com/s/c42158cdee23f25489cd. These ambiguous contigs were removed from the genomes before building the databases.

Creation of curated microbial genome databases: A custom Kraken database was build and contained 5751 different genomes (Supplementary Table S4). It included archaeal (251), bacterial (5166), fungal (225), protozoan (73), and viral (35) genomes from which ambiguous sequences had been removed (see above), and the human reference genome GRCh38.p7. Low complexity regions in this library of genomes where masked using dustmasker from the C++ Toolkit version 12 [2] with standard settings. Masked bases where then converted to N. The database was build using Kraken (0.10.6-unreleased) with the standard parameter for k=31 and M=15 [3]. A script for generating the kraken database is available from github (https://github.com/philDTU/endoPublication). We also generated a BLAST database, which contained the same genomes as the kraken database and was build using makeblastdb.

Illustration of the importance of filtration and classification of curated microbial genome databases – See, Figure at figshare: https://figshare.com/s/a282670f1405eae232df. Classifying unfiltered quality trimmed reads using a Kraken database composed of non-curated microbial reference genomes and the human reference results in the identification of many reads that are mapping to *Toxoplasma gondii* and *Plasmodium vivax*. In fact, these

genomes recruit more reads than the causing agent *Enterococcus faecalis*. The lowest common ancestor approach in Kraken cannot assign it to a higher level, because the highest-weighted-root-to-leaf-path is stronger for the contaminated contigs than the human reference. The classification is improved when human DNA sequences are filtered out prior to classification and a cleaned reference database is used.

Evaluation of a threshold for generating a curated microbial genome database: To build the database we used a conservative filtering of ambiguous sequence fragments (contigs/scaffolds). We deemed a sequence as ambiguous in the cases where we detected at least one BLAST hit that was affiliated with a different genus than the stated genus of the query sequence (under conditions: e-value ≤ 1e-6; query coverage ≥ 70%). To evaluate the impact of such a conservative filtering we analysed the BLAST hits in more detail.
For each sequence fragment we determined the ratio between the number of negative BLAST hits (query genus ≠ subject genus) and all BLAST hits. A low ration would indicate a higher chance for the sequence fragment to be ambiguous. We filtered the potentially ambiguous sequences by different ratio levels between 0.05 and 1.00. For the 0.05 threshold, a sequence fragment was flagged as ambiguous when the query genus accounted for less than 5% of all BLAST hits. When we set the threshold to 1.00, we have the most conservative case with only one BLAST hit outside of the query genus being required to classify a sequence as ambiguous.
The impact of the threshold varied from microorganism to microorganism. For the organisms relevant to our study the impact was minimal. The bacterial genomes in the database are mostly closed and return no fragments below 10.000 bases with the exception of small plasmids. A decline over the threshold was observed for *Toxoplasma gondii*. Our method performed though well on the *Toxoplasma gondii* sample from the Doan *et al*. study (see main text), mainly because even in the most conservative case we removed only around 1% of the genome sequence. Additionally, it is to expect that most of the k-mers in those ambiguous sequences were linked to a higher taxonomic level in kraken. The results from this analysis are summarized in a table available from figshare (https://figshare.com/s/b2db263f05db3b571aed).

Comparison of bioinformatics workflows: We analyzed a simulated mock community (MetaSimHC, by Peabody *et al*, 2015) compose of 11 microorganisms, and compared the results from our Kraken+Bracken workflow (outlined in Fig. 2) with the results obtained through analyses performed by Peabody and colleagues using 12 metagenomic classifiers [4]. The results from this comparison are available from figshare (https://figshare.com/s/07dfa4f99e663fc79164).

4. *Classification of reads using Kraken and Bracken*
We employed Kraken to assign a read to its respective taxon. To classify a set of reads, Kraken splits each read into all possible k-mers and searches for perfect matches in its database [3]. The database consists of a hash-table matching each k-mer to all genomes in the database it is found in. One k-mer can be assigned to multiple organisms and is then assigned to the lowest common ancestor. For example, if a k-mer is found in two differed *Bacillus* species the k-mer is assigned to the genus instead of species level. The taxon label for the whole read is then evaluated based on the taxon labels for the individual k-mers. Kraken returns read counts for each label in the taxonomy tree, thus one will obtain counts on all taxonomic levels. Species abundance is then estimated using Bracken [5].

Reads are re-distributed to the desired level by Bayesian probability estimation. Further, only species with at least 5 reads assigned by kraken are considered for taking up new reads. This is to minimize the number of false positive species.

5. *Classification of reads using BLASTN*
In addition, we used BLASTN to align the reads to genomes in a BLAST database. The BLAST database contained the same genomes as the kraken database. Hits needed at least a p-value of 1e-6 and a minimal query coverage of 80.

6. *Metagenomic assemblies*

Metagenomic shotgun reads were assembled, after human-affiliated reads had been removed, using SPAdes (3.10.1). The assembled contigs were submitted to the Bacterial Analysis Pipeline [6], which is described below.

7. Mapping of metagenomic shotgun reads to reference genomes

To further examine the presence of the potential causing microbial agent in the vitreous body fluid, we mapped metagenomic shotgun reads to the reference genome sequence of the microorganism that was identified to be the most abundant using the Kraken and BLAST analyses. Reads classified to at least the genus level of the most abundant species where extracted and mapped using the BBmap suite.

Whole genome sequence analysis

Reads were adapter trimmed and filtered for phiX reads using BBduk. In addition, we removed reads mapping to the masked human reference genome hg19 using the BBmap suite The masked human reference was generated by the BBsuit developer (https://drive.google.com/file/d/0B3llHR93L14wd0pSSnFULUlhcUk/edit?usp=sharing). Masked regions are of low entropy, multiple repeat or conserved regions from other fungal and plants. Masking a total of around 1.4% from the reference.

The high-quality reads where assembled using the SPAdes assembler [7] in careful mode with k-mer sizes 21, 33, 55, 77, 99, and 127. For each assembly, contigs smaller than 5 kb where aligned to the non-redundant nucleotide collection (nt) database from NCBI using BLASTn. Contigs that were larger than 1kb and exhibited a strong association to the same organism where kept in the final assembly. A quality assessment of the assemblies was performed using QUAST [8] (Supplementary Table S7). The genome sequence assemblies were analysed using the Bacterial Analysis Pipeline [6]. To determine the taxonomic identity of isolates, the pipeline utilizes a k-mer based approach [9]. Subsequently, a genomic MLST-typing is performed based on the allele and sequence profiles from PubMLST [10]. Antimicrobial resistance genes are detected using ResFinder with a minimal sequence identity of 90% and minimal resistance gene length coverage of 60% [11].
The average depth of coverage for the genome assemblies ranged between 88 times and 198 times. The number of contigs per assembly ranged in between 19 and 37 for *E. faecalis* and 44 to 74 for *S. epidermidis*. The total number of assembled bases and GC content was close to the median reference length and GC content reported at NCBI. 3 Mb and a GC content of 37.3% for *E. faecalis* and 2.4 Mb for *S. epidermidis* with a GC content

of 31.9%. The N50s were between 300k and 350k for *E. faecalis* and between 94k and 156k for *S. epidermidis* (Supplementary Table S7).

For the samples for which isolates & WGS assemblies were obtained, the metagenomic shotgun reads of the respective sample were mapped to the genome assembly of the isolate using the BBmap suite.

Targeted PCR analysis

As another independent assessment for the most abundant organism in a vitreous sample we employed targeted PCR assays to detect the two most frequently identified bacteria, *Staphylococcus epidermidis* and *Enterococcus faecalis*. For the detection of *S. epidermidis* the species-specific primer pair SE705-1 (5'-ATC AAA AAG TTG GCG AAC CTT TTC A-3') and SE705-2 (5'-CAA AAG AGC GTG GAG AAA AGT ATC A-3') was used [12]. For the detection of *E. faecalis* the species-specific primer pair *ddl*-E1 (5'-ATC AAG TAC AGT TAG TCT-3') and *ddl*-E2 (5'-ACG ATT CAA AGC TAA CTG-3') was used [13]. PCR products were preferentially detected in the samples in which the respective bacteria were also represented by high metagenomic read counts, as well as for some samples were these organisms were represented by only a few metagenomic read counts. We obtained no PCR products from samples for which these organisms were neither detected in the metagenomics analysis, nor the cultivation based analyses like MALDI-TOF and WGS. In case of the *S. epidermidis*-specific PCR, we also did not obtain PCR products from vitreous samples that contained the closely-related *S. hominis*. More details about the targeted PCR assays are available at https://figshare.com/s/0e8a98f436f07efc4dd5.

References

1. Altschul, S. F., Gish, W., Miller, W., Myers, E. W. & Lipman, D. J. Basic local alignment search tool. *J Mol Biol* **215,** 403–410 (1990).
2. Morgulis, A., Gertz, E. M., Schaffer, A. A. & Agarwala, R. A fast and symmetric DUST implementation to mask low-complexity DNA sequences. *J Comput Biol* **13,** 1028–1040 (2006).
3. Wood, D. E. & Salzberg, S. L. Kraken: ultrafast metagenomic sequence classification using exact alignments. *Genome Biology* **15,** 1–12 (2014).
4. Peabody, M. A., Van Rossum, T., Lo, R. & Brinkman, F. S. L. Evaluation of shotgun metagenomics sequence classification methods usingin silico and in vitro simulated communities. *BMC Bioinformatics* 1–19 (2015). doi:10.1186/s12859-015-0788-5
5. Lu, J., Breitwieser, F. P., Thielen, P. & Salzberg, S. L. Bracken: estimating species abundance in metagenomics data. *PeerJ Computer Science* **3,** e104 (2017).
6. Thomsen, M. C. F. *et al.* A Bacterial Analysis Platform: An Integrated System for Analysing Bacterial Whole Genome Sequencing Data for Clinical Diagnostics and Surveillance. *PLoS ONE* **11,** e0157718 (2016).
7. Bankevich, A. *et al.* SPAdes: A New Genome Assembly Algorithm and Its Applications to Single-Cell Sequencing. *Journal of Computational Biology* **19,** 455–477 (2012).
8. Gurevich, A., Saveliev, V., Vyahhi, N. & Tesler, G. QUAST: quality assessment tool for genome assemblies. *Bioinformatics* **29,** 1072–1075 (2013).
9. Hasman, H. *et al.* Rapid Whole-Genome Sequencing for Detection and Characterization of Microorganisms Directly from Clinical Samples. *Journal of Clinical Microbiology* **52,** 139–146 (2014).
10. Larsen, M. V. *et al.* Multilocus Sequence Typing of Total-Genome-Sequenced Bacteria. *Journal of Clinical Microbiology* **50,** 1355–1361 (2012).
11. Zankari, E. *et al.* Identification of acquired antimicrobial resistance genes. *Journal of Antimicrobial Chemotherapy* **67,** 2640–2644 (2012).
12. Martineau, F., Picard, F. J., Roy, P. H., Ouellette, M. & MG, B. Species-specific and ubiquitous DNA-based assays for rapid identification of Staphylococcus epidermidis. *Journal of Clinical Microbiology* **34,** 2888–2893 (1996).
13. Dutka-Malen, S., Evers, S. & Courvalin, P. Detection of glycopeptide resistance genotypes and identification to the species level of clinically relevant enterococci by PCR. *Journal of Clinical Microbiology* **33,** 24–27 (1995).
